# Tuning the acidity of halloysite by polyionic liquid to develop an efficient catalyst for the conversion of fructose to 5-hydroxymethylfurfural

**DOI:** 10.1038/s41598-023-34876-4

**Published:** 2023-05-11

**Authors:** Samahe Sadjadi, Soheila Yaghoubi, Xuemin Zhong, Peng Yuan, Majid M. Heravi

**Affiliations:** 1 Gas Conversion Department, Faculty of Petrochemicals, Iran , Polymer and Petrochemical Institute, PO Box 14975-112, Tehran, Iran; 2 Department of Chemistry, School of Physic and Chemistry, Alzahra University, PO Box 1993891176, Vanak, Tehran, Iran; 3 CAS Key Laboratory of Mineralogy and Metallogeny/Guangdong Provincial Key Laboratory of Mineral Physics and Materials, Guangzhou Institute of Geochemistry, Chinese Academy of Sciences, Guangzhou, 510640 China; 4 School of Environmental Science and Engineering, Guangdong University of Technology, Guangzhou, 510006 China

**Keywords:** Chemistry, Catalysis, Organic chemistry

## Abstract

In an attempt to prepare a low-cost and efficient acidic heterogeneous catalyst for the conversion of fructose to 5-hydroxymethylfurfural under mild reaction conditions, the acidity of halloysite was improved by covalent grafting of an acidic polyionic liquid. More precisely, halloysite was first vinyl functionalized and then polymerized with vinyl imidazole and 2-acrylamido-2-methylpropanesulfonic acid. The tangling imidazole rings were further converted to acidic ionic liquids by treating them with chlorosulfuric acid. UV–Vis spectroscopy and Hammett equation confirmed that conjugation of acid polyionic liquid resulted in the increase of the acidity of halloysite. Investigation of the efficiency of the catalyst for the synthesis of 5-hydroxymethylfurfural and optimization of reaction variables showed that 5-hydroxymethylfurfural was yielded in 97.8% after 30 min under the optimum conditions, i.e. catalyst loading of 20 wt% at 70 °C. Notably, the catalyst was highly reusable and it could be reused for at least seven reaction runs with insignificant loss of its activity. Furthermore, this catalyst could also promote the conversion of sucrose and maltose to give moderate yields of 5-hydroxymethylfurfural.

## Introduction

The use of renewable resources has been considered a solution to the shortage of conventional energy resources and environmental pollution^1^. There are various types of renewable energies, such as wind, ocean, solar and geothermal energy, among which bioenergy has attracted considerable attention. In this class of renewable energy, biomass, such as agricultural waste, plants, wood, etc. is converted to electricity or fuels, referred to as biofuels^2^. Taking advantage of biofuels into account, many attempts have been devoted to developing biofuels with competing features with gasoline. In this line, four generations of biofuels have been promoted^2–4^. The first generation of biofuels consists of alcoholic fuels, such as bio-ethanol. As this class of biofuels has a lower energy density in comparison with conventional fuels and suffers from various technical issues, a second generation of biofuels that is based on furan-based compounds has been developed. Gratifyingly, furan-based biofuels have higher energy density and more importantly, they can be produced from inedible resources, mostly lignocellulosic biomass^5^. Formation of furan-based biofuels consists of two main steps, i.e. conversion of lignocellulosic biomass to the platform compounds, such as 5-hydroxymethylfurfural (HMF)^6–8^ following some chemical reactions, such as hydrodeoxygenation to form biofuels, such as 2,5-dimethylfuran.

Synthesis of HMF^9–11^ from carbohydrates as the initial stage towards the production of furan-based biofuels^12^ is of great importance^13,14^. Moreover, as this key compound can be utilized for the synthesis of other chemicals ^15^, such as levulinic acid^16,17^, many research groups attempted to disclose efficient protocols for the synthesis of HMF by developing different catalytic systems. To date, various catalysts, such as Lewis acid catalysts^18^ and H-form zeolites^19^ have been developed for this acid-catalyzed reaction. In this regard, acidic ionic liquids (ILs) can also be considered as potential candidates. One of the advantages of these organic salts^20–27^ is that they can be readily tuned and designed for a specific purpose^28^. Acidic ILs also can be prepared by chlorosulfonation of the organic moiety^29^. More interestingly, ILs can be polymerized to form polyionic liquids, PILs, which benefits from myriad of ILs. Alternatively, conventional polymers can be converted to PILs via chemical modifications. Furthermore, it is possible to support ILs/PILs on various supporting materials, such as natural clay minerals via facile chemical reactions, to increase the catalytic performance and stability of ILs/PILs. Natural clay minerals, which benefit from availability, low cost, and chemical and thermal stability are economic and environmentally benign supporting materials. Some clay minerals, such as halloysite (Hal) exhibit excellent efficiency for catalysis^30–38^. Hal that is an aluminosilicate with cylindrical morphology and oppositely electrically charged surfaces has been extensively applied for the immobilizations of catalysts^38^. Hal also possesses acidic characteristics. However, its acidity is not strong enough to be able to catalyze the conversion of carbohydrates to HMF.

**Figure 1:**
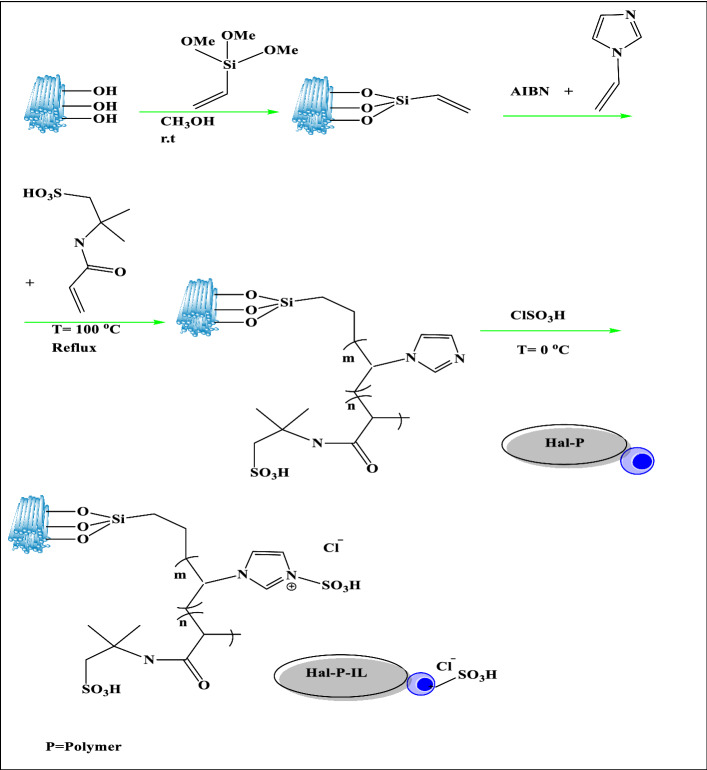
Synthetic procedure of the catalyst.

Hence, to modify the acidic features of Hal, in this study, an acidic PIL was covalently grafted onto Hal through the polymerization of vinyl-functionalized Hal with vinyl imidazole and 2-acrylamido-2-methylpropanesulfonic acid, followed by the conversion of imidazole rings to IL by reacting with chlorosulfuric acid, Fig. 1. This nanocomposite, which contains both acidic ILs and –SO_3_H functional groups in its backbone was then applied as a catalyst for the conversion of fructose to HMF. The effects of the reaction variables for this reaction have been investigated and the optimal reaction conditions were obtained. Moreover, the recyclability and stability of the catalyst was affirmed and a comparative study was accomplished to show the merit of the catalyst.

## Result and discussion

### Characterization of Hal-PIL

**Figure 2:**
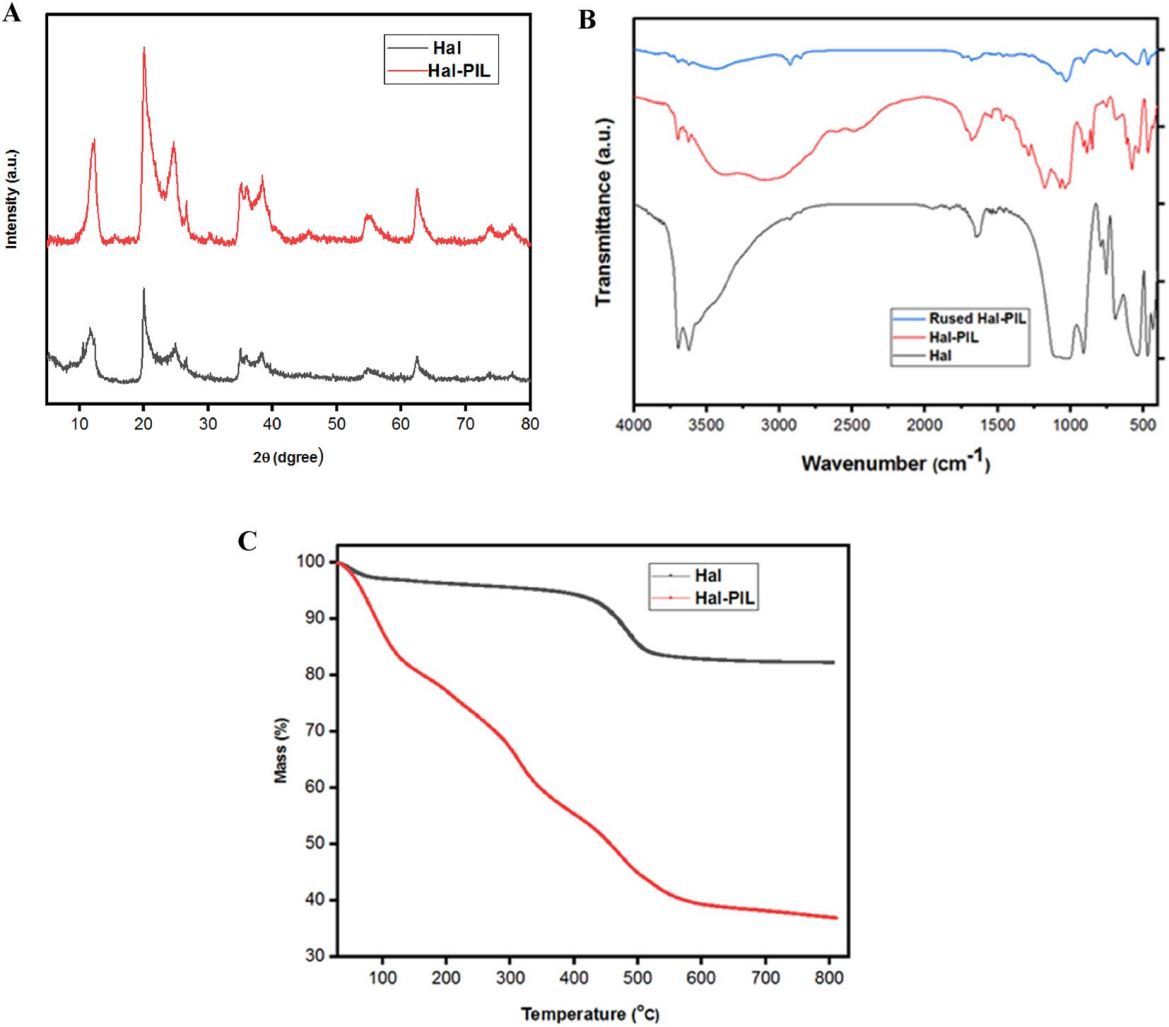
(**A**) XRD patterns of Hal and Hal-PIL, (**B**) FTIR spectra of Hal, Hal-PIL and reused Hal-PIL and (**C**) Thermograms of Hal and Hal-PIL.

To confirm the stability of Hal in the course of polymerization and conjugation of PIL, XRD pattern of Hal-PIL was compared with Hal. As displayed in Fig. 2A, the two recorded XRD patterns were very similar and displayed all of the characteristic peaks of Hal, i.e. the peaks appeared at 2θ = 12.3°, 18.8°, 20.6°, 25.2°, 36.7°, 39.0°, 56.3° and 62.5°^38^, were detected in the XRD pattern of Hal-PIL. Noteworthy, the peaks exhibited no displacement, indicating the stability of Hal structure. The only difference between the two patterns is the intensity of the peaks, which was reduced due to the grafting of PIL on Hal.

Conjugation of PIL on Hal was also approved via FTIR spectroscopy. As depicted in Fig. 2B, the FTIR spectrum of Hal showed the characteristic absorbance bands of Hal at 536 cm^-1^ (Al–O–Si vibration), 746 cm^-1^ (stretching vibration of Si–O), 795 cm^-1^ (symmetric stretching of Si–O), 1035 cm^-1^ (Si–O stretching), 1102 cm^-1^ (perpendicular Si–O–Si stretching), 1658 cm^-1^ (weak stretching and bending vibrations of water), 3697 cm^-1^ and 3629 cm^-1^ (interior –OH). These peaks were also detectable in the FTIR spectrum of Hal-PIL. This result along with the results of XRD analysis confirms the structural stability of Hal after the functionalization with PIL. It is also worth mentioning that the absorbance bands at 1690 and 1710 cm^-1^ in the FTIR spectrum of Hal-PIL are indicative of –C=N and –C=O functionalities in the backbone of the polymeric moiety. Moreover, the absorbance band of O=S=O functionality (1030 cm^-1^) overlapped with that of Hal. These results show that PIL functional groups were successfully grafted on Hal support.

To estimate the loading of PIL on Hal, the thermograms of Hal-PIL and Hal were recorded and compared. As shown in Fig. 2C, the two thermograms are considerably different. More accurately, Hal as a clay mineral was highly thermally stable and in its thermogram, only loss of water and dihydroxylation at elevated temperature (~ 500 °C) can be detected. In the thermogram of Hal-PIL, however, an additional weight loss (~ 30 wt%) in the range of 200–360 °C was observed that is ascribed to the decomposition of PIL. In fact, this result indicated that loading of the high content of PIL on Hal is achievable through using the described synthetic procedure.

EDS analysis was conducted to further confirm the formation of Hal-PIL. As illustrated in Fig. S1, carbon, nitrogen, aluminium, silicon, chlorine, oxygen and sulfur atoms were detected in the structure of Hal-PIL. The presence of carbon, nitrogen, chlorine and sulfur atoms in the EDS analysis approved the conjugation of PIL on Hal. Although EDS is a semi-quantitative analysis, the high content of sulfur atom in Hal-PIL (19.43 wt%) confirms high loading of acidic –SO_3_H moiety in the catalyst. Elemental mapping analysis of Hal-PIL in Fig. S2 affirms that the polymeric moiety has been formed uniformly on Hal and all of the representative elements of PIL have been dispersed homogeneously.

**Figure 3:**
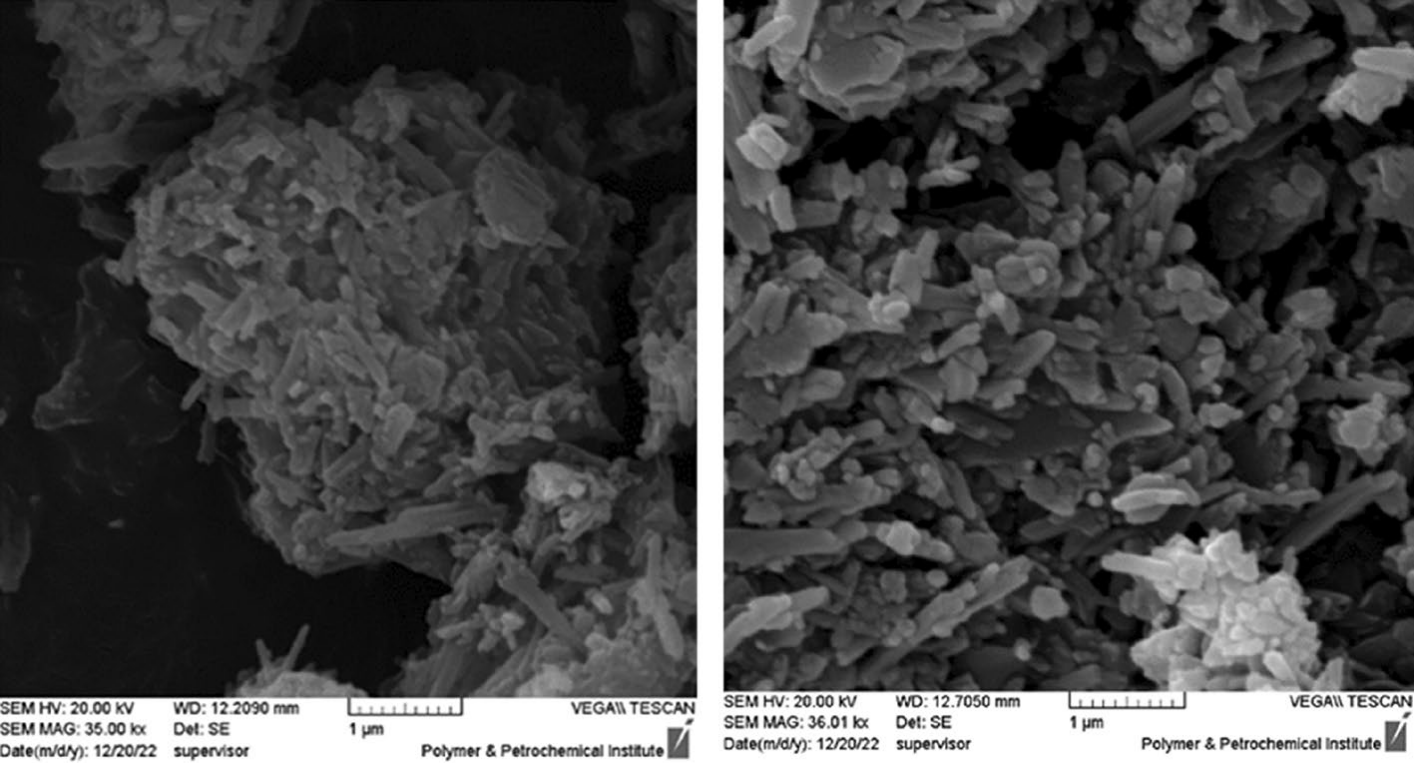
SEM images of Hal-PIL.

Morphological study of Hal-PIL was conducted by obtaining SEM images of Hal-PIL. As illustrated in Fig. 3, in Hal-PIL, the tubes of Hal were observable, affirming that the instinct tubular morphology of Hal was preserved upon functionalization. However, in Hal-PIL, Hal tubes were packed and formed aggregates. This issue is ascribed to the presence of PIL. In fact, the electrostatic interactions between PIL-decorated Hal tubes can be responsible for the formation of aggregates.

To appraise the effect of the conjugation of PIL on the specific surface area of (S_BET_) Hal, S_BET_ of Hal-PIL was obtained and compared with Hal. The results confirmed that upon the introduction of PIL on Hal, S_BET_ of Hal reduced from 48 m^2^/g to 21 m^2^/g, affirming the surface coverage of Hal by PIL. Nitrogen adsorption–desorption isotherm of Hal-PIL, Fig. S3, showed type II with H3 hysteresis loops according to the IUPAC classification.

To assess the acidity of Hal-PIL, UV–Vis spectroscopy and Hammett equation (Eq. 1) was applied. In this method, 4-nitroaniline was utilized as a basic indicator [I] and its UV–Vis spectrum was recorded at λ_max_ = 382 nm, Fig. S4. UV–Vis spectrum of I in the presence of Hal-PIL was also obtained and the decrement of absorbance was observed due to the formation of protonated I, [IH]^+^. Obtaining the adsorption of [I] and [IH]^+^, Hammett function, H°, can be calculated using Eq. (1).

1 $$ {\text{H}}^\circ = {\text{p}}K\left( {\text{I}} \right)_{{{\text{aq}}}} + {\text{log}}\left[ {\text{I}} \right]/\left[ {{\text{IH}}} \right]^{ + } $$

where p*K*(I) is the pK_a_ value for I.

Apart from Hal-PIL, H° value was also estimated for pristine Hal to elucidate whether introduction of PIL on Hal affected the acidity. As the results show, Table 1, H° value for Hal-PIL is lower than that of Hal, implying higher acidity of the former. This result approves that grafting of PIL on Hal leads to the increase of its acidity.

**Table 1:** Value of Hammett function (H) for Hal and Hal-PIL.

Entry	IL	A_max_	[I]%	[HI]^+^ %	H_°_
1	Blank	0.98	100	0	–
2	Hal-PIL	0.55	56	44	1.00
3	Hal	1.02	68	32	1.31

A_max_: absorbance at λ_max_.

### Catalytic activity of Hal-PIL for the synthesis of HMF

#### Optimization of reaction variables

Dehydration of fructose to HMF is highly affected by reaction conditions. In this line, parameters, such as reaction time, temperature and loading of the catalyst are of great importance. Hence, optimization of these variables is essential for achieving a high yield of HMF.

### Effect of Hal-PIL loading

**Figure 4:**
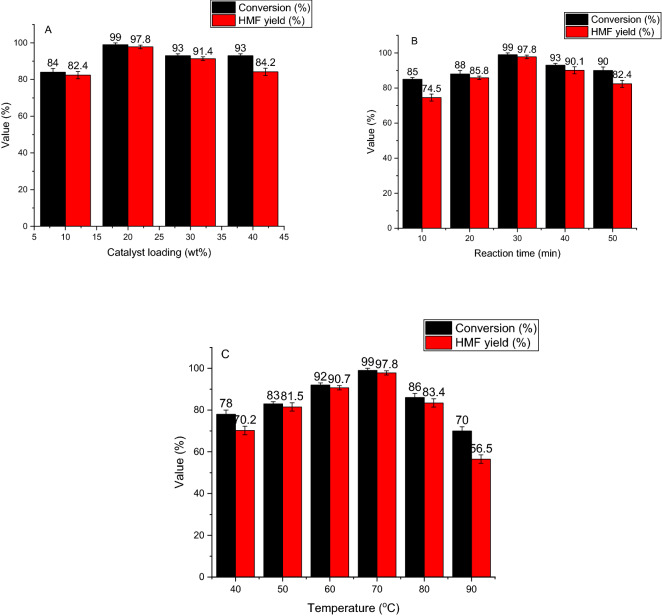
(**A**) Investigation of the effect of the catalyst loading on the fructose conversion and yield of HMF in dehydration of fructose. Reaction conditions: temperature: 70 °C, t = 30 min, solvent: DMSO, (**B**) Investigation of the effect of the reaction time on the fructose conversion and yield of HMF in dehydration of fructose. Reaction conditions: temperature: 70 °C, catalyst loading 20 wt%, solvent: DMSO and (**C**) Investigation of the effect of the reaction temperature on the fructose conversion and yield of HMF in dehydration of fructose. Reaction conditions: catalyst loading 20 wt%, t = 30 min, solvent: DMSO.

To investigate the effect of Hal-PIL loading on the HMF yield, fructose dehydration was conducted at 70 °C in DMSO as a solvent in the presence of different loadings of Hal-PIL (10–40 wt%) and HMF yield of each reaction was measured after 30 min. Comparison of the yields of HMF, Fig. 4A, indicated the crucial role of Hal-PIL loading on the reaction. As depicted, in the presence of only 10 wt% of the catalyst, 82.4% HMF was obtained, and upon increasing Hal-PIL to 20 wt%, HMF yield increased to 97.8%. The fact that only low loading of the catalyst can lead to the formation of a high yield of HMF can be ascribed to the moderate acidic feature of Hal-PIL. More precisely, -SO_3_H functional groups on the PIL can efficiently catalyze dehydration of fructose to HMF. As shown in Fig. 4A, the use of higher content of Hal-PIL (30 and 40 wt%) had a converse effect and led to the decrement of HMF yield. In fact, with the increment of the catalyst amount, the number of acidic sites will increase, which can catalyze the formation of HMF and also its condensation to humins^39^ and formation of side products that consequently results in the decrease of HMF yield.

### Effect of reaction time

The effect of reaction time on the HMF yield was studied. In this line, dehydration of fructose to HMF was performed in the presence of 20 wt% Hal-PIL in DMSO at 70 °C. The HMF yield of the reaction was measured in various reaction times (10–50 min) and the results were compared. As displayed in Fig. 4B, with the increase of the reaction time from 10 to 30 min, the HMF yield gradually increased from 74.5 to 97.8%. In fact, increase of the reaction time led to the increase of the contact of fructose to the catalytic active sites and the formation of a higher yield of HMF. Upon further increment of the reaction time to 50 min, a converse trend was observed and HMF yield started to decrease. This issue is quite expectable as longer contact time to acidic sites of the catalyst can trigger side reactions and the formation of humins.

### Effect of reaction temperature

Dehydration of fructose to HMF is a temperature-dependent process. Hence, investigation of the effect of this parameter is imperative for the optimization of the reaction conditions. To this purpose, synthesis of HMF was accomplished in DMSO using Hal-PIL (20 wt%) for 30 min at different temperatures (40, 50, 60, 70, 80 and 90 °C). The yields of HMF at different temperatures are illustrated and compared in Fig. 4C. As shown, an increase in the reaction temperature from 40 to 70 °C resulted in the increase of HMF yield from 70.2 to 97.8%, which is because the increase of the reaction temperature can improve the kinetic energy and effective contact of the substrate and the catalytic active sites. However, elevating the reaction temperature to 80 and 90 °C had a converse effect and led to the decrease of HMF yield to 83.4% and 56.5%, respectively. This issue can be justified by the increase of the possibility of formation of humins and also occurring of side reactions at higher temperatures.

### Effect of the reaction solvent

The effects of three different solvents, including water, ethanol and butanol on the dehydration of the fructose under the optimized reaction conditions were also investigated and compared with that of DMSO. The results indicated that use of water, ethanol and butanol was less effective than DMSO and led to lower yields of HMF due to the formation of by-products (HMF yields in water, ethanol and butanol were 65%, 60% and 69% respectively).

### Catalyst recyclability in dehydration of fructose to HMF

**Figure 5:**
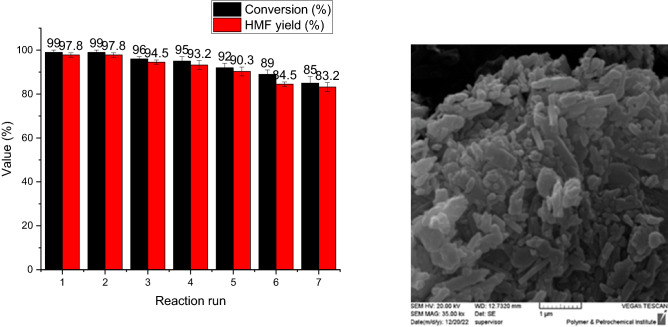
(**A**) The results of recyclability of Hal-PIL for the synthesis of HMF from dehydration of fructose under the optimum conditions and (**B**) SEM image of the recycled catalyst after the last run of recycling.

The recyclability of Hal-PIL for the synthesis of HMF from dehydration of fructose under the optimum conditions was examined for seven consecutive runs. Gratifyingly, Hal-PIL exhibited high recyclability and preserved its catalytic activity for the second run, Fig. 5A. For other runs, also, only slight a loss of the catalytic activity of the catalyst was observed, and HMF yield decreased to 83.2% after seven runs.

The FTIR spectra of the recycled Hal-PIL recovered from the last run of the reaction, and the fresh catalyst are shown in Fig. 2B. It can be observed that the intensity of some bands, such as the band appeared > 3000 cm^-1^ in the spectrum of the reused catalyst decreased remarkably.

To appraise the possible morphological change upon recycling, SEM image of reused Hal-PIL after the last run of recycling was obtained, Fig. 5B. As displayed, recycling of Hal-PIL for several runs did not cause significant change in its morphology and packed Hal tubes are observable in the SEM image of the reused catalyst. Furthermore, to appraise the leaching of –SO_3_H groups, the reused catalyst after the last run was characterized via EDS analysis. Comparison of the content of S atom in the fresh (11.05 wt%) and the reused catalyst (10.1 wt%) indicated that leaching of –SO_3_H groups, which were conjugated on Hal covalently, was insignificant.

### Reaction mechanism

**Figure 6:**
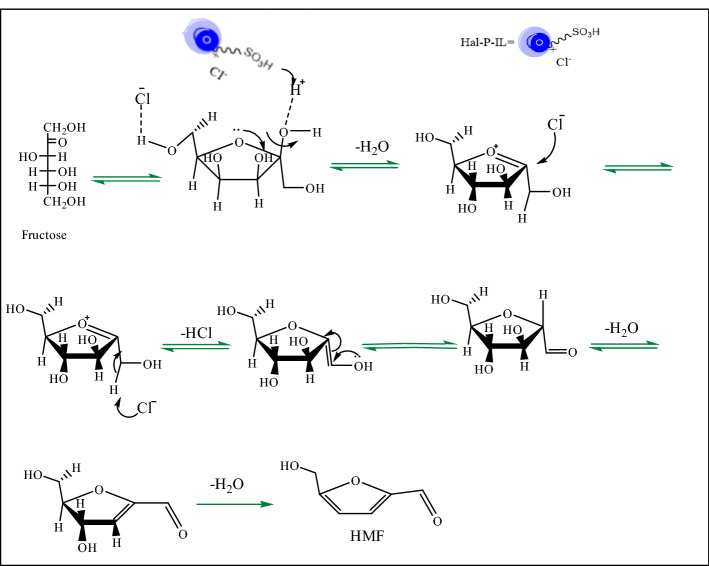
Pictorial mechanism of conversion of fructose to HMF in the presence of Hal-PIL.

A proposed reaction mechanism for the preparation of 5-hydroxymethylfurfural by dehydration of fructose is shown in Fig. 6. According to the literature^40^, in the first step, a hydrogen bond is formed between Hal-PIL and the -OH functional group of fructose and one molecule of water will be removed. Next, the anion in the structure of Hal-PIL will react to form an enolic intermediate, which then tolerates tautomerization followed by loss of water molecules to form HMF.

### Synthesis the catalyst

#### Generality of the protocol

**Figure 7:**
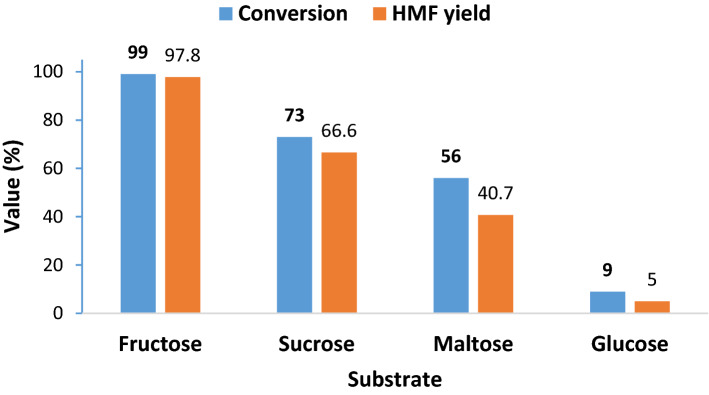
The catalytic activity of Hal-PIL for the synthesis of HMF using different substrates. Reaction conditions: temperature: 70 °C, catalyst loading 20 wt%, t = 30 min.

The results of the study of the catalytic activity of Hal-PIL for the conversion of fructose to HMF confirm that Hal-PIL can be considered as an efficient heterogeneous catalyst for promoting dehydration of fructose to HMF and led to the formation of this key compound in 97.8% under mild reaction conditions. As the conversion of other carbohydrates to HMF is more challenging, it was expected that lower yields were obtained by using other substrates. To appraise this issue, the catalytic activity of Hal-PIL for the conversion of three more substrates, i.e. sucrose, maltose and glucose was examined. As illustrated in Fig. 7, Hal-PIL could catalyze the conversion of sucrose to yield HMF in 66.6%. As in the case of sucrose, which is composed of glucose and fructose units, conversion to HMF requires additional steps, i.e. hydrolysis of sucrose and further isomerization of glucose to fructose, this reaction was more tedious compared to the conversion of fructose to HMF that only consists of the dehydration reaction. Hence, a lower yield can be justified. Similarly, in the case of maltose, which is a disaccharide formed from two glucose units, an even lower yield was obtained under the optimum reaction conditions. Among the examined carbohydrates, glucose led to the lowest yield.

The study of the catalytic activity and recyclability of Hal-PIL for dehydration of fructose confirmed that this catalyst with a low content (20 wt%) could catalyze this key reaction in a short reaction time (30 min) to furnish HMF in 97.8% yield. To further elucidate the merit of this heterogeneous catalyst, its activity for this model reaction was compared with a range of catalysts that have been reported in the literature (Table 2). Notably, this comparison cannot highlight the superiority of a specific protocol as the reaction conditions are different, but can shed light on the activity of the catalyst to some extent. As tabulated, a wide range of catalysts, from bio-based catalysts to composite catalysts have been reported for this reaction. Obviously, bio-based catalysts, such as Hal-PIL and cellulose-based catalyst (Table 2, entry 9) benefit from some advantages, such as availability and environmentally benign nature. Synthetic catalysts, such as metal–organic frameworks (Table 2, entry 3) and mesoporous silica (Table 2, entries 4 and 5) and dendritic catalysts (Table 2, entry 8) may suffer from time-consuming, tedious or multi-step synthetic protocols as well as the use of toxic solvents and reagents, which render them costly and less environmentally-friendly. Compared to L-Proline derived ionic liquids, Table 2, entry 10, Hal-PIL exhibited higher catalytic activity and can give HMF in higher yield in shorter reaction time. Similarly, the comparison of the reaction conditions and activity of Hal-PIL and FPIL, which is a PIL-based catalyst (Table 2, entry 13) affirmed the superior activity of the former. In the case of the catalysts that are composed of heteropolyacids, such as PW_12_-ILs-C4-HNS (Table 2, entry 11), Hal-PIL as a metal-free catalyst showed higher activity at lower temperature and a shorter reaction time. Therefore, it is concluded that Hal-PIL as a bio-based heterogeneous catalyst can be recognized as a promising composite for the dehydration of fructose to HMF.

**Table 2:** Dehydration of fructose using various catalysts.

Entry	Catalyst	Temp. (ºC)	Time (min)	Reaction conditions	Yield (%)	Ref.
1	Hal-PIL	70	30	DMSO	97.8	–
2	Mesoporous TiO_2_	130	2	Microwave , DMA-LiCl	82.3	^41^
3	MIL-101(Cr)-SO_3_H	120	60	DMSO	90	^42^
4	SBA-SO_3_H	160	70	MIBK/2-butanol,	55	^43^
5	SBA-15-SO_3_H-10	120	60	–	~ 81	^44^
6	PS-Tet^a^-SO_3_H Brønsted acid	150	120	DMSO	86.85	^45^
7	Fe_3_O_4_@SiO_2_-SO_3_H	100	120	DMSO	93.1	^46^
8	SO_3_H-dendrimer-SiO_2_@Fe_3_O_4_	100	60	Solvent free	92	^47^
9	Cell-G3-SO_3_H^b^	110	45	Acetone:water(2:1)	96	^48^
10	L-Proline derived ionic liquids	90	50	–	73.6	^49^
11	PW_12_-ILs-C4-HNS^c^	100	120	DMSO	93.7	^50^
12	Si-3-IL-HSO_4_	130	30	–	63.0	^51^
13	FPIL^d^	120	60	–	88.3	^52^
14	SPAN^f^	140	180	Water/1,4-dioxane	71	^53^

^a^polystyrene functionalized 5-amino-1H tetrazole (PS-Tet).

^b^3G dendrimer on the surface of modified cellulose.

^c^heteropolyacids stabilized IL-modified organosilica hollow nanosphere.

^d^bifunctional polymeric ILs (Immobilizing Cr^3+^ with SO_3_H-functionalized solid PIL).

^f^Sulfonated polyaniline.

## Conclusion

In summary, a novel acidic heterogeneous catalyst, Hal-PIL, was developed through grafting of acidic PIL groups to halloysite. Study of the performance of Hal-PIL for the conversion of fructose to HMF approved high activity of Hal-PIL due to the increase in instinct acidity of halloysite. Moreover, the investigation of the effects of the reaction variables showed that HMF can be achieved in 97.8% within 30 min using 20 wt% Hal-PIL in DMSO at 70 °C. It is also worth mentioning that Hal-PIL could be readily recovered and recycled for seven reaction runs with slight loss of its activity after each run. In addition, the investigation of the catalytic performance of Hal-PIL for conversion of other carbohydrates to HMF indicated that this catalyst could catalyze reaction of sucrose and maltose to give HMF in moderate yield.

### Experimental

#### Materials and instruments

The list of materials and instruments are mentioned in supporting information.

### Preparation of poly ionic liquid-functionalized Hal (Hal-PIL)

Preparation of Hal-PIL was achieved through a three-step procedure. The detail of each step is as follow:

### Functionalization of Hal with vinyltriethoxysilane (Hal-V)

Hal (4 g) was added to toluene (60 mL) and stirred for 10 min to furnish a homogeneous suspension. Next, vinylterimetoxysilane (4 mL) was added, and then the reaction mixture was refluxed for 24 h at 110 °C. The percipitaye was obtained via centrifugation, washed several times with toluene, and then dried at 70 °C for 24 h.

### Preparation of polymer-functionalized Hal (Hal-P)

Hal-V (2 g) was dispersed in a mixture of deionized water and ethanol (20 mL) with a ratio of 1:2 and then a solution of AIBN (0.1 g in ethanol) was introduced and the mixture was mixed for 20 min at room temperature. Subsequently, the monomers, i.e. VI (2 g) and AMPS (2 g) were added and the mixture was refluxed for 24 h under Ar atmosphere. Afterward, the precipitate was collected and washed with deionized water and dried at 60 °C for 24 h.

### Conversion of polymer to polyionic liquid: synthesis of Hal-PIL

Hal-P (2 g) was suspended in CH_2_Cl_2_ (16 mL) and stirred at 0 °C for 14 min. Then, chlorosulfuric acid (0.7 mL) was dropped slowly into the aforementioned suspension and the mixture was stirred for 24 h at room temperature. After this step, the precipitate, Hal-PIL, was separated, rinsed with dichloromethane (CH_2_Cl_2_) three times, and dried at 60 °C for 24 h.

### HMF synthesis

Hal-PIL (0.02 g) was added to a solution of fructose (0.1 g) or other starting materials in DMSO (4 mL). The mixture was then stirred at 70 °C for 30 min to form yellowish HMF. After the completion of the reaction, Hal-PIL was collected, washed with DMSO repeatedly and dried at 60 °C overnight. The detail of HMF purification and calculation of HMF yield is provided in the supporting information.

## Supplementary Information

Supplementary Information.

## Data Availability

The datasets used and/or analyzed during the current study are available from the corresponding author on reasonable request.
