# Supplementary material for: Tuning the acidity of halloysite by polyionic liquid to develop an efficient catalyst for the conversion of fructose to 5-hydroxymethylfurfural
Source: Sci Rep. 2023 May 11;13:7663. doi: 10.1038/s41598-023-34876-4 (PMC10175272; doi:10.1038/s41598-023-34876-4)
Supplement: Supplementary file 1 — Supplementary Information. [file 41598_2023_34876_MOESM1_ESM.docx]

**Supplementary information**

**Tuning the acidity of halloysite by polyionic liquid to develop an efficient catalyst for the conversion of fructose to 5-hydroxymethylfurfural**

Samahe Sadjadi^1*^, Soheila Yaghoubi^2^, Xuemin Zhong^3^, Peng Yuan^4^**,** Majid M. Heravi*^2^

**Materials and instruments**

Halloysite (Hal), vinyltriethoxysilane (97%), vinyl imidazole (VI, >99%), azobisisobutyronitrile (AIBN, 98%), 2-acrylamido-2-methylpropanesulfonic acid (AMPS, 99%), chlorosulfuric acid (97%), dichloromethane (CH_2_Cl_2_), ethanol (>99%), dimethyl sulfoxide (DMSO, >99%), fructose (>99%), glucose (>99%), maltose (>99%), cellulose, all provided from Sigma-Aldrich, were applied for the synthesis of Hal-PIL and investigating its catalytic activity for the synthesis of HMF.

The analyses used to confirm the formation of Hal-PIL were X-ray diffraction (XRD, Rigaku Ultima $Ⅳ$ with Cu-Kα). Infrared spectroscopy (FT-IR, BRUKER TENSOR 35 spectrophotometer 65 with a scan time of 1s and spectral resolution of 2 cm^-1^ by using potassium bromide (KBr) pellets. Thermogravimetric analysis (TGA, METTLER TOLEDO, under O_2_ atmosphere and ramp rate of 10 °C/min), Scanning electron microscope (SEM) and energy-dispersive X-ray spectroscopy (EDS, VEGAII TESCAN device, equipped with QX2, RONTEC energy dispersive X-ray analyzer). The acidity of the catalyst and pristine halloysite was also measured using UV-Vis spectroscopy and the Hammett equation. The specific surface areas (S_BET_) of Hal and Hal-PIL were estimated using Brunauer-Emmett-Teller (BET) method using BELSORP MINI II, BEL instrument with pre-heating at 150 °C for 3 h.

**HMF yield**

The obtained HMF was purified through a known procedure [^49^](#_ENREF_49). Briefly, water saturated with NaCl (9 mL) was added to the reaction mixture to form organic and aqueous phases. HMF in the organic phase was separated via Rotary evaporation, while the low content of HMF in the aqueous phase was obtained via the addition of diethyl ether (35 mL), following distillation. Quantitative analysis and measurement of HMF yield were conducted using Gas chromatography (GC) and High-performance liquid chromatography (HPLC).

GC analysis was performed using Agilent 6890 gas chromatograph with a flame ionization detector (FID) and G&W HP-5 ms GC column. For this analysis, N_2_ was utilized as a carrier gas at a split ratio of 100:1. Moreover, inlet and detector temperatures were set at 275 °C and 285 °C respectively.

For HPLC analysis, Agilent 1200 Series apparatus equipped with a Brisa LC2 C18 column (5 µm, 25 $\times$ 0.46) operated at 35 ºC based on the external standard was applied. To identify the compounds, pure HMF was used and its retention time was compared with the sample. An auto-sampler (Agilent G1329A) was utilized to enhance the reproducibility. The eluent with the flow a rate of 1 mL min^-1^ was a mixture of acetonitrile to water with a volumetric ratio of 40:60.

HMF yield was simply achieved using Equation 1, where, Mole (F) is the initial moles of fructose.

$$HMF yield \left( \% \right)=\frac{Mole (HMF)}{Mole\left( F \right)}\times100\% Eq. 1$$

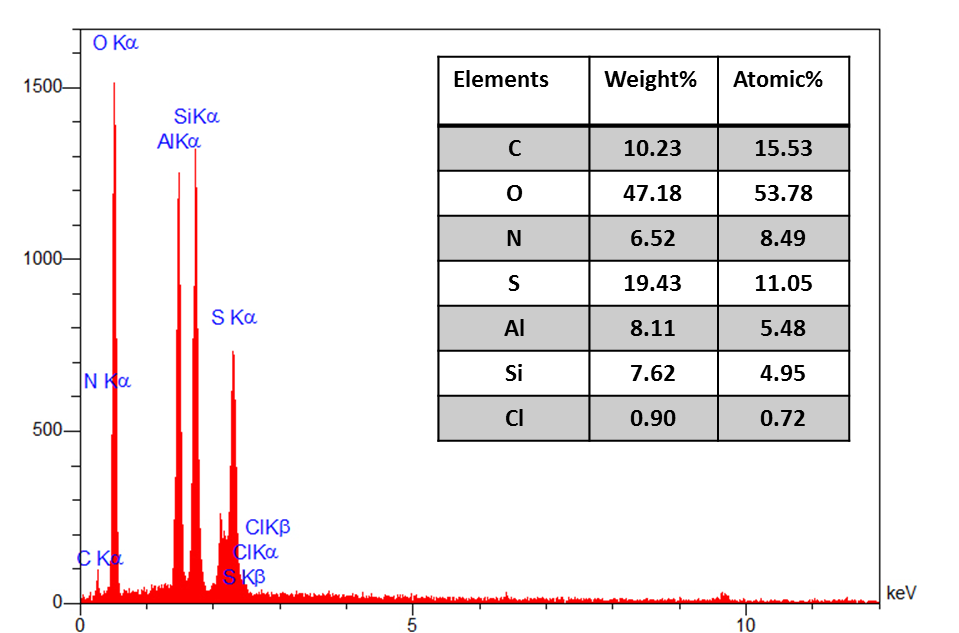


**Figure S1.** EDS analysis of Hal-PIL.


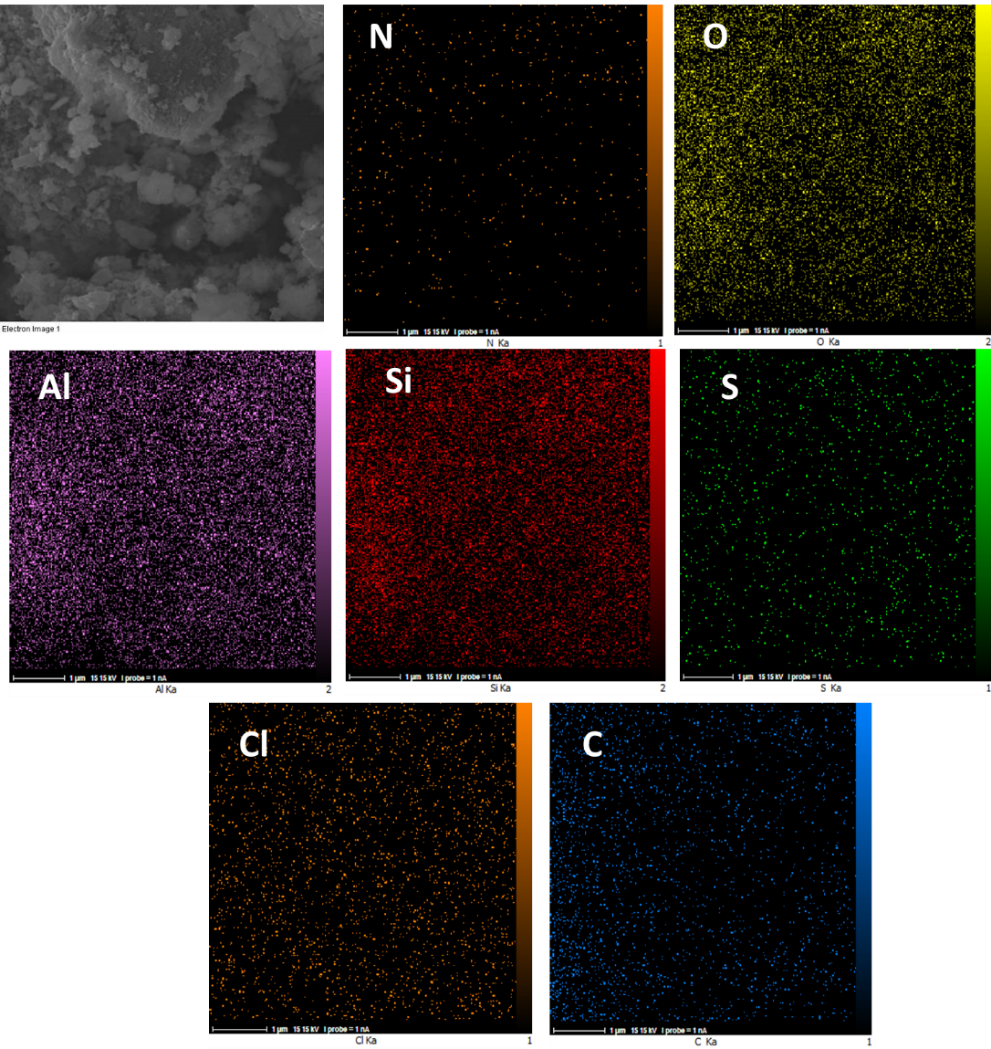


**Figure S2.** Elemental mapping analysis of Hal-PIL.

**Figure S3.** Nitrogen adsorption–desorption isotherm of Hal-PIL


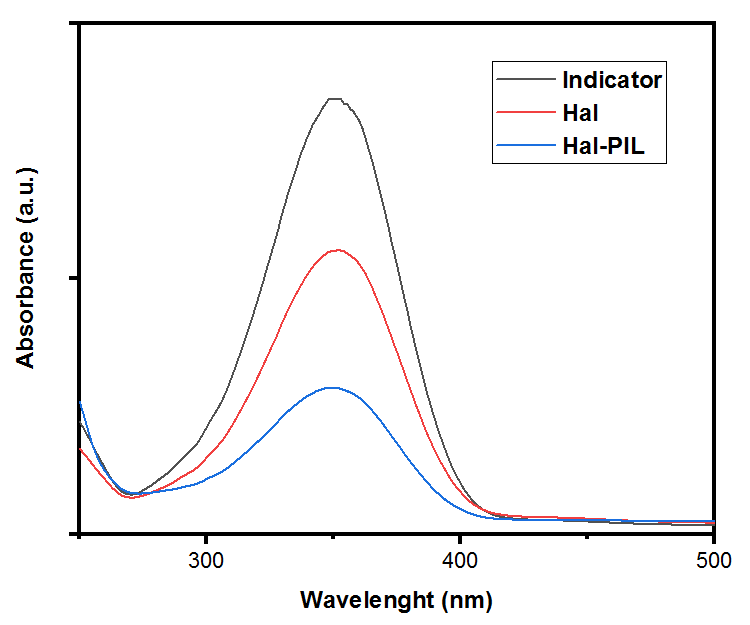


**Figure S4**. Hammett plot of Hal and Hal-PIL using basic indicator 4-nitroaniline.
